# The impact of internet use on the health status of the older adults in China: a chain mediating model of social interaction and physical exercise

**DOI:** 10.3389/fpubh.2025.1603406

**Published:** 2025-08-01

**Authors:** Jiangwei Hu, Haoxian Mai, Chunyun Tan, Yingying Du

**Affiliations:** ^1^School of Chinese Literature and Media, Hubei University of Arts and Science, Xiangyang, Hubei, China; ^2^School of Journalism and Communication, Guangdong University of Foreign Studies, Guangzhou, Guangdong, China

**Keywords:** internet use, health status, social interaction, physical exercise, aged, China

## Abstract

**Background:**

As China’s population aging intensifies, older adults face growing health risks. Internet use, social interaction, and physical exercise significantly impact their health. This study aims to explore the underlying mechanisms of these variables on older adults health.

**Methods:**

This study used data from the 2021 Chinese General Social Survey (CGSS), involving 3,150 urban and rural older adults aged 60–98. The dependent variable was health status, measured by a composite score of self-assessed health, physical health, and mental health. Internet use served as the independent variable. Social interaction and physical exercise were the mediators. A chain mediation model linking internet use, social interaction, physical exercise, and health status was created. The relationships between these factors were tested using Pearson correlation analysis, multiple linear regression, and Bootstrap.

**Result:**

Internet use demonstrated a significant positive impact on older adults’ health status (*β* = 0.086, *p* < 0.001), which supports the hypothesis that Internet use improves health outcomes. The total indirect effect of the chain mediation model was 0.0165 (95% CI = [0.0103, 0.0239]). Specifically, the indirect effect of the path Internet use → social interaction → health status was 0.0080 (95% CI = [0.0037, 0.0131]), the indirect effect of the path Internet use → physical exercise → health status was 0.0077 (95% CI = [0.0035, 0.0126]), and the indirect effect of the path Internet use → social interaction → physical exercise → health status was 0.0009 (95% CI = [0.0004, 0.0016]). Social interaction and physical exercise partially and chain-mediate the relationship between Internet use and health status in older adults.

**Conclusion:**

This study reveals that internet use significantly impacts older adults’ health both directly and indirectly through enhanced social interaction and physical exercise. The government should promote internet adoption among older adults, especially in underserved rural and low-income areas, by enhancing infrastructure and offering inclusive digital training. Additionally, financial support and policy incentives should be provided to encourage the development of age-friendly applications focused on social interaction and sports. Policymakers should advance the integration of internet services with geriatric health policies to enhance overall well-being.

## 1 Introduction

In 2024, the proportion of the global population aged 65 and over will be about 10.5%, compared with 15.6% in China, well above the global average. China has the world’s largest older adults population and is experiencing a faster ageing process than the global average (1). Since the late 1970s, China’s population ageing process has accelerated, with an annual growth rate of about 3.2 percent; while this process has taken more than 45 years in developed countries, it has taken only about 27 years in China, and it is expected that the aging trend will intensify in the long term (2). As China’s population ages, an increasing number of Chinese seniors are facing health risks, particularly in the domain of mental health (3, 4). Meanwhile, the continuous advancement of media technology has led to an expansion of media forms from traditional newspapers, radio, and television to new media platforms, such as the Internet. Modern new media, particularly the Internet, are impacting and changing people’s lifestyles and behaviors. The China Internet Network Information Centre (CNNIC) reports that, as of June 2024, there were over 157 million Internet users aged 60 and above, accounting for 14.3% of all Internet users (5). The Internet penetration rate of the older adults population is increasing significantly, and the level of Internet use is deepening. For the older adults, the use of the Internet for online services such as information search and entertainment experience has become a basic need for enjoying life in old age and reintegrating into society (6), and at the same time, it is a new factor affecting the health of the older adults.

Although several studies have explored the links between Internet use and health in older adults, further investigation is required to elucidate the mechanisms underlying these links. The hypothesis that Internet use by older people enables them to have more social contacts and participate in various social activities, thereby promoting social group interaction, has been postulated (7). The process of social group interaction has the potential to enhance the well-being of Chinese older adults by increasing their pleasure, reducing loneliness, and alleviating depression (8, 9). Consequently, social interaction may be a pivotal mediating factor in this relationship. Additionally, within the Chinese context, the ‘Healthy China Strategy’ explicitly identifies sport as a pivotal initiative in addressing the challenges posed by aging. One study found that Internet use may influence physical exercise (10), which in turn may promote physical and mental health in older adults (11), and that physical exercise may also be an important mediator. Therefore, what are the pathways through which Internet use affects the health status of older people, and what are the specific mechanisms of action? Further in-depth discussions are needed. The present study, therefore, focuses on the impact of Internet use on the health status of older adults, with an emphasis on the mediating roles of social interaction and physical exercise, aiming to provide theoretical and practical references for improving the health of older adults and promoting healthy aging.

## 2 Literature review and research hypotheses

### 2.1 Internet use and health status of older adults

Researchers have conducted a large number of empirical studies on the impact of Internet use on the health of older adults. In general, health includes a variety of dimensions, such as physical health and mental health. The existing literature indicates a close correlation between Internet use and multidimensional health in older adults (12). With regard to mental health, research has shown that the Internet can alleviate social isolation and loneliness in older adults (13). Yuan’s research indicates that older individuals in Shanghai who frequently use the Internet are less likely to experience psychological distress, particularly those with chronic conditions (14). Moreover, studies have demonstrated that older individuals who use the Internet are 33% less likely to suffer from depression compared to those who do not (15). In terms of physiological health, Internet use has been found to improve the health of older adults and reduce the incidence of high blood pressure and heart disease in older age groups (16, 17). In a longitudinal study based on data on aging in the United Kingdom, Xavier et al. found that older British people who regularly used the Internet had a higher propensity to prevent cancer (18). Concurrently, the utilization of the Internet has been observed to potentially mitigate the risk of developing dementia and cognitive decline (19), thereby promoting healthier behaviors (20).

### 2.2 Social interaction, physical exercise, and health in the older adults

Social interaction can be defined as the material and spiritual exchanges between individuals under specific social and historical conditions (21). A substantial body of research has demonstrated that older adults who participate in social activities exhibit superior cognitive functioning and perceived mental health compared to those who do not (22, 23). Yuasa et al. utilized cross-sectional data to examine the impact of social capital on mental health among older adults and discovered significant associations between informal social interactions and formal group participation and health variables (24). Concurrently, extensive research has consistently shown that physical exercise is positively correlated with perceived health, physical health, and mental health (25, 26). According to Leith and Taylor’s review of 81 studies on the connection between exercise and mental health that were published between 1979 and 1989, regular exercise is important for fostering mental health (27). Moreover, older adults who participate in physical exercise exhibit notably superior physical, psychological, and social health, as well as higher overall health scores and improved physical condition, compared to their inactive counterparts (28).

### 2.3 Internet use, social interaction, and physical exercise

As older people age, their social interactions gradually decrease, and their sense of isolation increases significantly. However, the Internet, as a platform for people to interact and communicate with each other online, supports the offline participation of older people in social activities. Atsushi’s research revealed that older adults who use the Internet on a daily basis are able to maintain stronger social connections, meet friends more often, and significantly enhance their subjective well-being compared to those who do not use the Internet at all (29). However, some studies have suggested opposing views, indicating that internet use may lead to the deterioration of individuals’ social networks and the weakening of interpersonal relationships (30). Emotional interactions in the virtual world of the internet have been found to replace deep real-world emotional exchanges, thereby reducing social interaction and participation in real life among older adults. There is also a correlation between Internet use and physical exercise. Some studies have indicated that older adults who frequently use the Internet tend to participate more in physical activities and are more likely to engage in moderate-to-vigorous physical exercise on a weekly basis (31). The more physically active older adults are and the more satisfied they are with their lives, the better their mental health will be (32). However, in a survey of Polish internet users over 50, Duplaga found that they are less inclined to participate in sports (33).

Secondly, there is a strong correlation between social interaction and physical exercise. The cohort effect suggests that individuals are no longer independent in their decisions and behaviors in social interactions and are directly influenced by the behaviors and characteristics of other groups in their environment (34). A study revealed that older adults who engage in emotional social interactions are 41% more likely to meet physical exercise standards (35). Furthermore, the construction of a favorable social environment and the establishment of a subjective emotional support system for older adults have been demonstrated to enhance the motivation of empty nesters to engage in physical exercise (36). Shephard et al. identified a suitable partner as a pivotal factor influencing an individual’s physical activity (37). Leith used the social interaction hypothesis to argue that social interactions with friends, colleagues, etc., during physical activity are pleasant (27).

### 2.4 Research hypothesis

**Figure 1:**
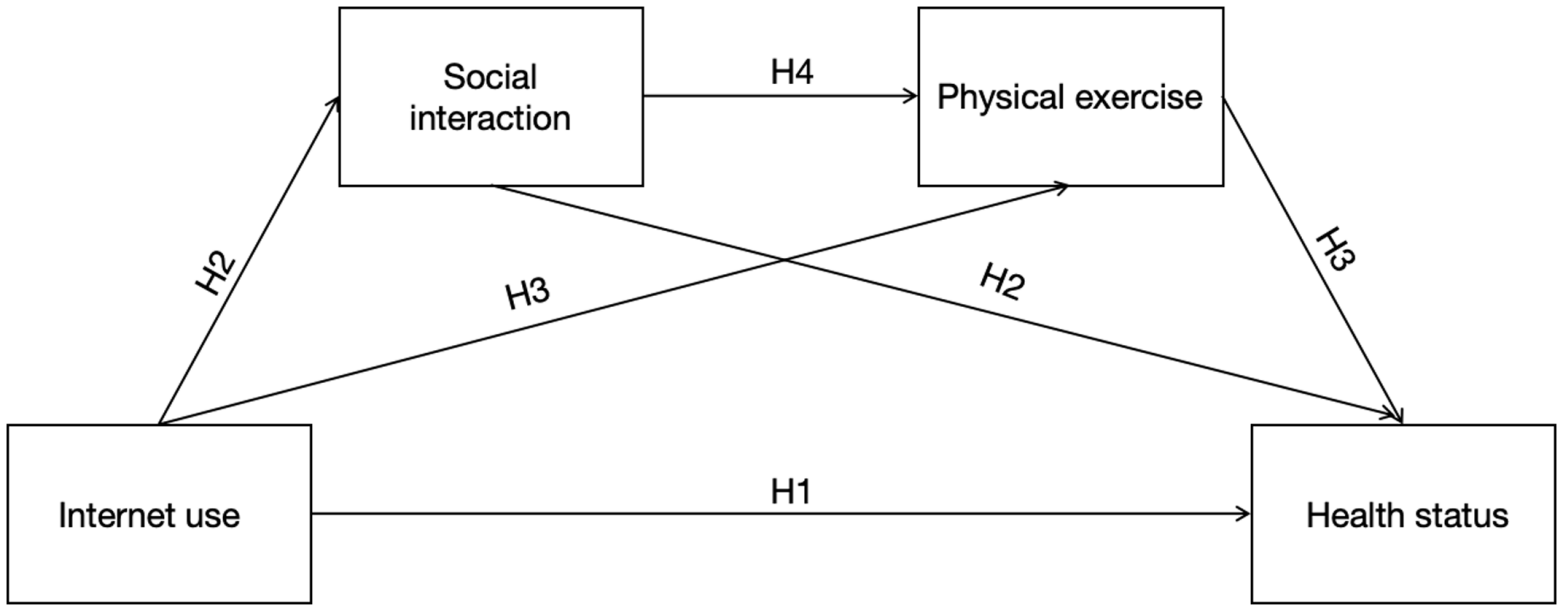
Assumption diagram. A hypothetical model of the relationship between internet use, social interaction, physical exercise, and health status.

Through the preceding discussion, it is evident that there are interactions among internet use, social interaction, physical exercise, and health status. However, the combined effects of these factors remain unclear. The roles of social interaction and physical exercise as mediators between internet use and health status require further empirical investigation. Moreover, the underlying mechanisms linking internet use to the health of older adults have not yet been systematically studied. Given the rapid advancement of internet technologies, the potential impacts of internet use on the health of older adults deserve increasing attention. The findings of this study can offer additional insights for future research on older adult health. Therefore, this study proposes a chain-mediated hypothetical model (Figure 1) to clarify the relationships among internet use, social interaction, physical exercise, and the health status of older adults, and presents the following research hypotheses:

*H1*: Internet use has a significant positive impact on the health status of older adults.

*H2*: Social interaction mediates the relationship between Internet use and the health status of older adults.

*H3*: Physical exercise mediates the relationship between Internet use and the health status of older adults.

*H4*: Social interaction and physical exercise are chained mediators in the relationship between Internet use and the health status of older adults.

## 3 Materials and methods

### 3.1 Data

The dataset utilized in this study originates from the 2021 China General Social Survey (CGSS), an ongoing cross-sectional survey that has been conducted since 2003, encompassing over 10,000 households across all provinces, autonomous regions, and municipalities directly governed by the central government in mainland China. The CGSS adheres to the ethical principles outlined in the Declaration of Helsinki and was approved by the ethics committees of Renmin University of China and the Hong Kong University of Science and Technology, including participant consent procedures. Since the data are publicly accessible and anonymized, this study does not require additional ethical approval or informed consent procedures. The data can be accessed at http://cgss.ruc.edu.cn/. The use of this data complies with the terms and conditions set by the database. The survey’s questionnaire data are comprehensive and authoritative, with 8,148 samples collected in 2021, offering a representative dataset and a robust foundation for this research. In conjunction with the questionnaire items, responses with incomplete answers were excluded as invalid, resulting in a final sample of 3,150 urban and rural older adults aged 60–98 years.

### 3.2 Dependent variable

This study examines the health status of older adults. The World Health Organization (WHO) defines health as “health not only as the absence of disease, but as a state of complete physical, mental, social, and moral well-being” (38). Health can be measured by objective and subjective indicators, and self-assessed health is a reliable indicator for evaluating health status, which is a subjective evaluation of the state of health based on the objective health status of an individual (39). Therefore, this study used the subjective assessment of health by older adults to measure health status. Drawing on the research idea of Yang et al. (2023) (40), health status was assessed based on respondents’ answers to questions regarding self-assessed health, physical health, and mental health in the CGSS. Respondents were asked, “How would you rate your current health status?” and were required to rate their health on a scale from 1 to 5 (1 = very unhealthy, 2 = somewhat unhealthy, 3 = fair, 4 = somewhat healthy, 5 = very healthy). Regarding physical health, respondents were asked, “How often have health problems affected your work or other daily activities in the past 4 weeks?” and were required to rate this on a scale from 1 to 5 (1 = always, 2 = often, 3 = sometimes, 4 = rarely, 5 = never). For mental health, respondents were asked, “How often have you felt depressed or down in the past 4 weeks?” and were required to rate this on the same scale (1 = always, 2 = often, 3 = sometimes, 4 = rarely, 5 = never). We consolidated the aforementioned questions to create a new variable termed ‘health status,’ which has been utilized in prior research to assess the influence of physical and mental health on the subjective well-being of older adults (41). Drawing from this reference, we calculated a composite score to evaluate the respondents’ health status, with scores ranging from 3 to 15, where higher scores denote better health. The Cronbach’s alpha value of this variable in this study is 0.76, indicating that this variable has high reliability.

### 3.3 Independent variable

The independent variable in this study is Internet use, based on the research framework proposed by Dong et al. (42). We primarily utilized a question from the CCSS2021 questionnaire: “How often did you use the Internet (including via mobile phone) in the past year?” Responses ranged from 1 to 5, indicating the frequency of Internet use from low to high: 1 = never, 2 = seldom, 3 = sometimes, 4 = often, and 5 = very often. Higher values reflect more frequent Internet use among older adults.

### 3.4 Mediating variables

The first mediating variable in this study is social interaction. The group nature of human social life determines that people inevitably form a variety of relationships with each other, primarily including relatives, friends, colleagues, and neighbors. Therefore, social interaction in this study refers to the positive interpersonal relationships formed between older adults and their close neighbors and relatives through mutual interaction and joint participation in social activities. Drawing on the research framework of Zhang et al. (43), four questions from the CGSS were selected to measure the degree of social interaction, focusing on respondents’ engagement in the following activities: gathering with relatives who do not live together, meeting with friends, social and recreational activities with neighbors (e.g., visiting each other’s homes, watching TV, eating together, playing cards, etc.), and social and recreational activities with other friends (e.g., visiting each other’s homes, watching TV, eating together, playing cards, etc.). In this study, reverse-scored items in the original questionnaire were processed accordingly, and the total scores of the four questions were reassigned according to the frequency logic (from lowest to highest). The total score of the four questions was used to measure the social interaction variable, ranging from 4 to 24, with higher scores indicating a higher degree of social interaction. The Cronbach’s alpha value for this variable in this study was 0.63, indicating that the reliability of this variable is within the acceptable range.

The second mediating variable in this study is physical exercise. Responses to the question “In the past year, how often did you participate in physical exercise during your free time?” in the CGSS2021 questionnaire were categorized as never, several times a year or less, several times a month, several times a week, and every day, based on Liu and Zhong (44). The recoded variable was assigned values ranging from 1 to 5, with higher values indicating more frequent physical activity among older adults.

### 3.5 Control variable

Drawing on previous studies, this study included residence, gender, age, education, marital status, socioeconomic status and political affiliation as control variables. Specifically, gender was coded as 0 for female and 1 for male; education was classified into five categories: primary education and below (1), primary education (2), junior high school education (3), senior high school education (4), and university education and above (5); place of residence was coded as 0 for rural areas and 1 for urban areas; political affiliation was recorded as 0 for non-Party members and 1 for CCP members; and marital status was recorded as 1 for married (including first marriage with a spouse, separated but not divorced, and remarried with a spouse) and 0 for single (including divorced, widowed, cohabiting, and unmarried); Socioeconomic status is an older person’s self-assessment of their socioeconomic status. The scale ranges from 1 (lower class) to 5 (upper class).

### 3.6 Common method deviation test

In this study, the common method bias test was conducted using Harman’s one-factor method, which showed that the first factor analyzed for all questions explained 21.95% of the variance, which was less than the 40% critical criterion, and more factors with eigenvalues greater than 1 were analyzed. Therefore, there is no significant common method bias in the study.

### 3.7 Statistical analysis

Initially, descriptive statistics and Pearson correlation analyses (*p* < 0.05 indicating significant correlation) were conducted on the sample variables. Subsequently, multiple linear regression analyses were conducted, controlling for gender, age, education, residence, marital status, socioeconomic status and political affiliation, to examine the relationships between Internet use, social interaction, physical exercise, and health status. It was hypothesized that all control variables would influence older adults’ health status. Finally, cascading mediation effects were analyzed using the PROCESS macro model developed by Hayes6 to investigate the mechanisms by which Internet use affects the health status of older adults. Bias-corrected bootstrapping was used, and a total of 5,000 bootstrap samples were extracted for the mediation test, with 95% confidence intervals not including 0 indicating a significant effect. The statistical analysis was performed using IBM SPSS Statistics 27.

## 4 Results

### 4.1 Descriptive statistic

As shown in Table 1, the mean score for the health status of the 3,150 older adults was 10.57 (SD = 2.987), indicating a relatively moderate level of health within this older population. For the independent variable of Internet use, the mean value was 2.34, suggesting that the majority of older respondents seldom used the Internet. Specifically, 53.4% of the respondents reported never having used the Internet, 8.1% rarely used it, 7.3% used it sometimes, 13.7% often used it, and 17.4% always used it. Regarding the mediating variables, social interaction was assessed with a mean score of 11.24 (SD = 4.724), reflecting moderate levels of social interaction among older persons. Physical exercise had a mean value of 2.79, indicating that the older adults had a relatively low level of sports participation. Finally, concerning the control variables, the average age of the older adults participants was approximately 71 years, with about 51.6% being women and 48.4% men. The educational attainment of the older adults was predominantly concentrated between primary and high school levels. Approximately 63% lived in urban areas and 37% in rural areas. Additionally, 15.6% were members of the Communist Party, and 75% were married. The majority of the older adults considered their socio-economic status to be in the lower middle range.

**Table 1:** Basic variable description statistic table.

Variable type	Variable	Mean/percentage	SD	Min	Max	Details
Dependent variable	Health status	10.62	2.98	3	15	Continuous variable
Independent variable	Internet use	2.34	1.616	1	5	Multi-categorical variable
	Never	53.40%				
	Rarely	8.10%				
	Sometimes	7.30%				
	Often	13.70%				
	Always	17.40%				
Mediating variables	Social interaction	11.24	4.724	4	24	Continuous variable
	Physical exercise	2.79	1.774	1	5	Multi-categorical variable
	Never	43.50%				
	Several times a year or less	7.60%				
	Several times a month	6.80%				
	Several times a week	10.40%				
	Every day	31.70%				
Control variable	Gender	0.48	0.5	0	1	Binary variables
	Female	51.60%				
	Male	48.40%				
	Education	2.63	1.133	1	5	Multi-categorical variable
	Illiterate	18.10%				
	Primary school	29.40%				
	Junior high school	29.20%				
	Senior high school	17.70%				
	College or above	5.60%				
	Residence	0.63	0.483	0	1	Binary variables
	Rural	37.00%				
	Urban	63.00%				
	Age	71.05	7.794	60	98	Continuous variable
	Marriage	0.75	0.433	0	1	Binary variables
	Married	75.00%				
	Unmarried	25.00%				
	Political status	0.16	0.363	0	1	Binary variables
	Party member	15.60%				
	Nonparty member	84.40%				
	Socioeconomic status	2.29	0.964	1	5	Multi-categorical variable
	Lower level	26.20%				
	Lower middle level	28.00%				
	Middle Level	37.70%				
	Upper middle level	7.20%				
	Upper Level	1.00%				

### 4.2 Pearson correlation analyses

Prior to the linear regression analysis, Pearson correlation analyses were conducted to examine the correlations among the variables (*p* < 0.05 indicating significant correlation). The results showed that the key independent variable, Internet use, was significantly and positively correlated with the health status of older adults (*r* = 0.205, *p* < 0.001), providing strong support for H1. Second, the mediator variables of social interaction and physical exercise were significantly and positively correlated with the health status of older adults; gender, education, residence, marital status, socioeconomic status and political status were significantly and positively correlated with the health status of older adults, while age was negatively correlated with health status. However, these correlations only indicate pairwise associations between the variables, necessitating further verification through regression analysis (Table 2).

**Table 2:** Results of Pearson’s correlation analysis between relevant study variables.

Variables	1	2	3	4	5	6	7	8	9	10	11
1. Health status	1										
2. Internet use	0.205^***^	1									
3. Social interaction	0.108^***^	0.118^***^	1								
4. Physical exercise	0.197^***^	0.234^***^	0.120^***^	1							
5. Gender	0.137^***^	0.006	−0.032	0.036^*^	1						
6. Education	0.236^***^	0.443^***^	0.033	0.284^***^	0.211^***^	1					
7. Residence	0.159^***^	0.199^***^	0.037^*^	0.244^***^	−0.078^***^	0.248^***^	1				
8. Age	−0.101^***^	−0.320^***^	−0.014	−0.066^***^	0.048^**^	−0.22^***^	0.044^*^	1			
9. Marriage	0.097^***^	0.12^***^	−0.01	0.042^*^	0.099^***^	0.141^***^	−0.003	−0.25^***^	1		
10. Political status	0.118^***^	0.164^***^	0.036^*^	0.156^***^	0.213^***^	0.309^***^	0.131^***^	0.143^***^	0.053^**^	1	
11. Socioeconomic status	0.262^***^	0.137^***^	0.099^***^	0.162^***^	0.005	0.159^***^	0.113^***^	0.057^**^	0.053^**^	0.154^***^	1

*p < 0.05; **p < 0.01; ***p < 0.001.

### 4.3 Multiple linear regression analysis

Multiple linear regression analyses were performed, with the health status of older adults as the dependent variable in all models (Table 3). Model 1 included control variables to assess the impact of gender, age, residence, education, marital status, socioeconomic status and political status on the health status of older adults. The results indicated that gender, age, residence, education, socioeconomic status and political status significantly affected the health status of older adults. Model 2 incorporated Internet use variables to examine their influence on health status. The results revealed a significant positive correlation between Internet use and health status (*β* = 0.086, *p* < 0.001), supporting Hypothesis 1. Model 3 introduced social interaction and physical exercise variables. The results showed that both social interaction (*β* = 0.066, *p* < 0.001) and physical exercise (*β* = 0.084, *p* < 0.001) were significantly and positively related to health status. After including social interaction and physical exercise in Model 3, the coefficient for Internet use decreased (*β* = 0.069, *p* < 0.001) but remained significant. This suggests that social interaction and physical exercise may mediate the relationship between Internet use and health status, providing a foundation for further exploration of the mediating mechanisms.

**Table 3:** Impacts of Internet use on Chinese older adults’ health status: based on a linear regression model.

	Model 1	Model 2	Model 3
Gender	0.118^***^	0.124^***^	0.125^***^
Age	−0.094^***^	−0.072^***^	−0.07^***^
Residence	0.116^***^	0.107^***^	0.092^***^
Education	0.115^***^	0.086^***^	0.075^***^
Marriage	0.032	0.032	0.034^*^
Political status	0.018	0.011	0.005
Socioeconomic status	0.231^***^	0.224^***^	0.211^***^
Internet use		0.086^***^	0.069^***^
Social interaction			0.066^***^
Physical exercise			0.084^***^
*R* ^2^	0.137	0.142	0.154
△*R*^2^	0.135	0.140	0.151
F(df)	71.250^***^	65.154^***^	56.983^***^
*N*	3,150	3,150	3,150

*p < 0.05; **p < 0.01; ***p < 0.001.

### 4.4 Mediation effect analysis

In this section, we further analyse the potential mechanisms by which Internet use affects the health status of older adults. In the literature review section, we describe potential relationships between Internet use, social interaction, physical exercise, and health status in older adults. In Pearson’s correlation analysis, we found a significant correlation between Internet use, social interaction, physical exercise, and health status. In the stratified regression, the coefficient of the influence of Internet use on the health status of the older adults decreased after adding the variables of social interaction and physical exercise, respectively. On this basis, we constructed a chain mediation model with health status of the older adults as the dependent variable, Internet use as the independent variable, and social interaction and physical exercise as the mediating variables. The bias-corrected Bootstrap method has been previously found to be more effective than the traditional Sobel test in testing the significance of mediating effects (45). The basic idea of this method is to resample the original data and extract the equal sample data to test the mediating effect. Therefore, in this study, Hayes was used to prepare the SPSS macro model 6 and 5,000 samples were extracted for the mediation test (46). If the 95% confidence interval of mediation does not contain 0, the mediation is significant; otherwise, the mediation effect is not significant.

**Figure 2:**
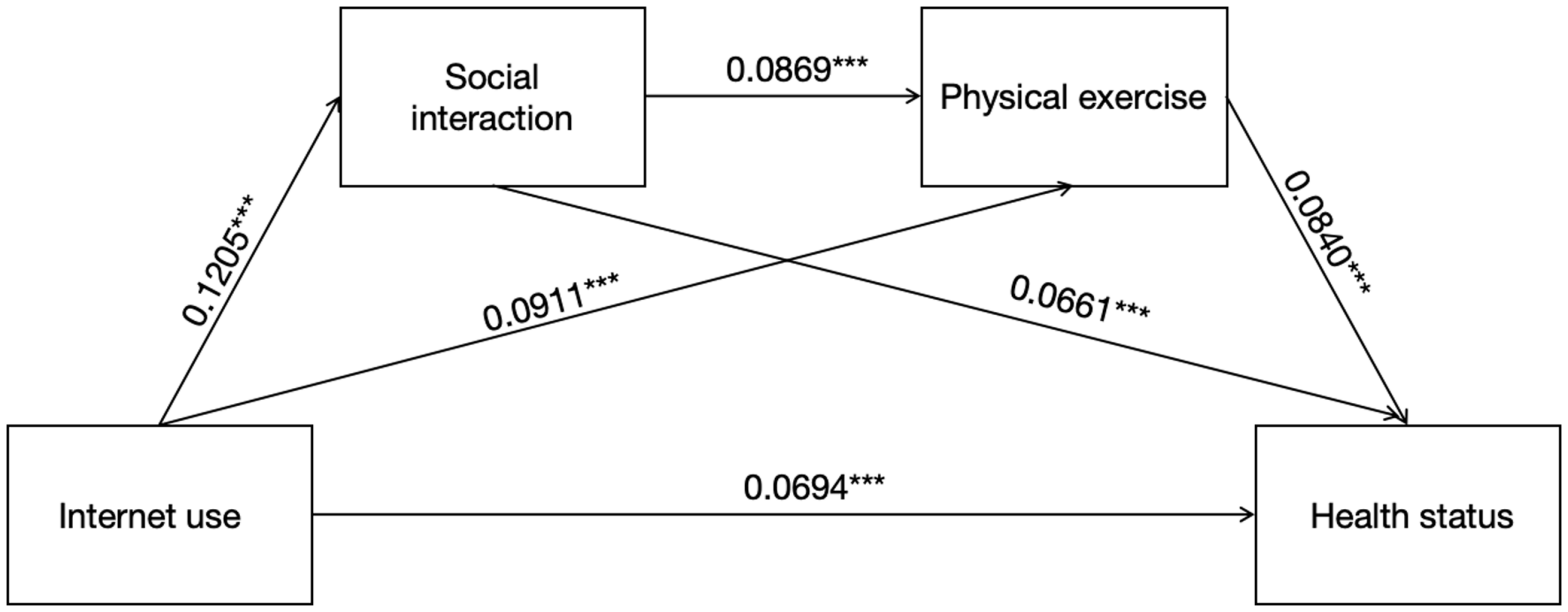
Chain mediation model of internet use and health status. ***p < 0.001. The numerical values illustrated in the figure correspond to the standardized regression coefficients for the respective.

As shown in Table 4 and Figure 2, the total indirect effect of the model was 0.0165 (95% CI = [0.0103, 0.0239]). The indirect effect mediated by social interaction was 0.0080 (95% CI = [0.0037, 0.0131]), with a confidence interval excluding 0, indicating that social interaction partially mediated the effect of Internet use on the health status of older adults. The indirect mediated effect of physical exercise was 0.0077 (95% CI = [0.0035, 0.0126]), with a confidence interval excluding 0, suggesting that physical exercise partially mediated the effect of Internet use on the health status of older adults. The indirect effect of social interaction and physical exercise as a chain mediator was 0.0009 (95% CI = [0.0004, 0.0016]), with a confidence interval excluding 0, confirming that social interaction and physical exercise as a chain mediator mediated the positive effects of Internet use on the health status of older adults, thus validating hypotheses 2, 3, and 4.

**Table 4:** Bootstrap test results for the chain mediation model.

Affect the path	Effect	BootSE	BootLLCI	BootULCL
Total effect	0.0859	0.0194	0.0478	0.1240
Direct effect	0.0694	0.0195	0.0312	0.1076
Total indirect effect	0.0165	0.0034	0.0103	0.0239
Internet use → Social interaction → Health status	0.0080	0.0024	0.0037	0.0131
Internet use → Physical exercise → Health status	0.0077	0.0024	0.0035	0.0126
Internet use → Social interaction → Physical exercise → Health status	0.0009	0.0003	0.0004	0.0016

### 4.5 Robustness test

We tested the robustness of the study in two ways. First, we tested whether the mediating effect was still significant across different demographic groups. Second, we replaced the key independent variables to see if the mediating effect would remain significant.

First, the chain mediation model was tested among male and female older adults (see Tables 5, 6). The results indicate that the partial and chained mediation of social interaction and physical exercise holds for both gender groups. Specifically, for older adult males, the partial mediating effect of social interaction was 0.0058 (95% CI = [0.0003, 0.0127]), the partial mediating effect of physical exercise was 0.0080 (95% CI = [0.0019, 0.0159]), and the chain mediating effect of social interaction and physical exercise was 0.0012 (95% CI = [0.0003, 0.0023]). For female older adults, the partial mediating effect of social interaction was 0.0103 (95% CI = [0.0035, 0.0185]), the partial mediating effect of physical exercise was 0.0064 (95% CI = [0.0007, 0.0139]), and the chain mediating effect of social interaction and physical exercise was 0.0006 (95% CI = [0.0001, 0.0015]).

**Table 5:** Bootstrap test results for the chain mediation model (based on the male older adult population).

Affect the path	Effect	BootSE	BootLLCI	BootULCL
Total effect	0.0839	0.0262	0.0325	0.1353
Direct effect	0.0689	0.0262	0.0175	0.1204
Total indirect effect	0.0150	0.0050	0.0063	0.0261
Internet use→ Social interaction →Health status	0.0058	0.0032	0.0003	0.0127
Internet use→ Physical exercise→ Health status	0.0080	0.0036	0.0019	0.0159
Internet use→ Social interaction → Physical exercise → Health status	0.0012	0.0005	0.0003	0.0023

**Table 6:** Bootstrap test results for the chain mediation model (based on the female older adult population).

Affect the path	Effect	BootSE	BootLLCI	BootULCL
Total effect	0.0852	0.0288	0.0288	0.1416
Direct effect	0.0679	0.0289	0.0113	0.1246
Total indirect effect	0.0173	0.0052	0.0080	0.0281
Internet use → Social interaction → Health status	0.0103	0.0038	0.0035	0.0185
Internet use → Physical exercise→ Health status	0.0064	0.0033	0.0007	0.0139
Internet use → Social interaction → Physical exercise → Health status	0.0006	0.0004	0.0001	0.0015

Second, we conducted robustness tests by replacing the independent variables. In the previous regression and mechanistic analyses, the measure of Internet use, which measures the frequency of Internet use among older adults, was a continuous variable. We replaced the question item with “In the last 6 months, have you gone online, including using various devices such as computers, cell phones, and smart wearables?” The answer was a binary categorical variable, and we recorded it as 0 for not having been on the Internet and 1 for having been on the Internet. Table 7 gives the results of substituting the independent variables without changing the other variables again. Social interaction and physical exercise continue to partially mediate and chain mediate the relationship between Internet use and health status among older adults. Therefore, the results of this study can be considered stable.

**Table 7:** Bootstrap test results for the chain mediation model (replacement of key independent variables).

Affect the path	Effect	BootSE	BootLLCI	BootULCL
Total effect	0.1409	0.0395	0.0635	0.2183
Direct effect	0.1069	0.0396	0.0292	0.1846
Total indirect effect	0.0340	0.0074	0.0203	0.0495
Internet use → Social interaction → Health status	0.0177	0.0056	0.0074	0.0293
Internet use → Physical exercise → Health status	0.141	0.0046	0.0059	0.0241
Internet use → Social interaction → Physical exercise → Health status	0.0022	0.0007	0.0010	0.0038

## 5 Discussion

This study utilized data from the 2021 China General Social Survey (CGSS) to investigate the associations between Internet use, social interaction, physical exercise, and health among older adults. Building on previous research regarding Internet use and the health status of older adults, this study delves deeper into the mechanisms by which Internet use influences their health, employing a chain-mediated model. Several significant conclusions emerge from this study. First, internet use demonstrated a significant positive impact on older adults’ health status (*β* = 0.086, *p* < 0.001), supporting Hypothesis 1. Second, the indirect effect for the path Internet use → social interaction → health status was 0.0080 (95% CI [0.0037, 0.0131]), indicating that social interaction partially mediates this relationship, thus supporting Hypothesis 2. Third, the indirect effect for Internet use → physical exercise → health status was 0.0077 (95% CI [0.0035, 0.0126]), confirming physical exercise’s partial mediating role and supporting Hypothesis 3. Finally, the chain mediation effect for Internet use → social interaction → physical exercise → health status was 0.0009 (95% CI [0.0004, 0.0016]), establishing the sequential mediating roles of social interaction and physical exercise, which supports Hypothesis 4.

Internet use has a positive impact on the health of Chinese older adults, consistent with most previous findings (47, 48). Our findings extend previous studies that simply categorized Internet use as “use or non-use” and concluded that Internet use has a positive effect on health status (49, 50). Specifically, the Internet provides a convenient channel for older people to acquire health knowledge. For example, using online health service websites to consult with specialists on disease diagnosis and treatment, searching for health/medical information through the Internet, and utilizing smartphones for health testing and management (51). Zhao et al. also found that older adults seek various types of health information on the Internet, including information about specific diseases, medications and treatments, nutrition and exercise, healthcare resources, disease symptoms, health promotion, health insurance, and health news or policies and health news or policy (52). Overall, the use of the Internet may increase older people’s access to health-related information, which may have a positive impact on their health status (53).

Furthermore, analyses of the mechanisms through which Internet use impacts the health of older adults indicate that both social interaction and physical exercise partially mediate the positive impact of Internet use on health, consistent with previous studies (54). Firstly, social interaction partially mediates the impact of internet use on health status. Internet use transcends temporal and spatial barriers, enabling older adults to stay informed about the daily lives of friends and relatives, thereby facilitating communication and potentially increasing the frequency of offline gatherings. Concurrently, internet usage expands social networks and enhances bridging social capital. This augmentation of overall social interaction elevates well-being and demonstrably improves psychological health, reducing symptoms of depression, anxiety, post-traumatic stress disorder (PTSD) (55, 56), and stress (57). Furthermore, the internet functions as a vital, bidirectional interactive medium for information dissemination. Older adults in China utilize it not only as passive recipients of health information but also as active disseminators via online platforms (e.g., blogs and short video platforms). By sharing personal health experiences, they foster community and facilitate health knowledge exchange, ultimately enhancing both social engagement and overall health. Secondly, physical exercise partially mediates the impact of internet use on health status. The proliferation of internet access provides individuals with readily available knowledge concerning health and physical activity, empowering them to identify appropriate forms of exercise (58). Additionally, the internet offers ubiquitous access to online training resources and professional coaching, enabling older adults to engage in physical activity with expert guidance (e.g., virtual fitness classes, and personalized exercise routines) (59). One study also found that during COVID-19, many public organizations posted information on the Internet about exercise programs and physical activity to prevent physical decline, and that older adults’ access to this information while using the Internet increased physical exercise (60). Maintaining regular physical exercise is an important mechanism for older adults to maintain their personal health and plays an important positive role in preventing chronic disease, maintaining physical function, and promoting overall health (61).

Additionally, a significant chain-mediated effect was identified between social interaction and physical exercise. This finding reveals a complex and progressive mechanism whereby internet use affects older adults health. It goes beyond a simple direct link, highlighting social interaction as a key bridge and physical exercise as a crucial behavioral converter. The internet offers convenient social platforms for the older adults. An active social life then acts as a catalyst for initiating and maintaining regular physical exercise, and together they improve health. By accessing the internet and utilizing digital platforms such as communication tools, social media, and online communities, the older adults can effectively overcome traditional social interaction constraints like geographical barriers, physical limitations, and shrinking social networks. This technological empowerment enhances both the frequency and quality of their social interactions. Video calls and instant messaging help maintain connections with distant friends and family, while interest-based communities and local forums enable the formation of new social relationships (62). Continuous online interaction alleviates loneliness, a significant health risk factor, and strengthens individuals’ connection to society through social recognition and emotional support. Notably, this enhanced social interaction becomes a key driver of physical exercise. Online social networks promote physical activity through several channels: Firstly, health knowledge and exercise experiences shared within these networks demonstrate positive examples and peer encouragement (63, 64), increasing the perceived value of exercise. Secondly, social media reduces the cost of organizing group activities, facilitating seamless transitions from online interactions (e.g., walking groups) to offline practices (e.g., square dance groups). Thirdly, virtual communities based on shared interests create a sense of belonging and responsibility, motivating members to maintain their social identity through continued participation in exercises. When social interaction translates into regular physical exercise, health benefits manifest through both physiological and psychological pathways. Physiologically, these benefits include improved cardiopulmonary function, enhanced musculoskeletal systems, and optimized metabolic indicators. Psychologically, endorphin release, increased self-efficacy, and strengthened social functioning form a positive feedback loop that helps sustain healthy behaviors. Overall, this also represents a plausible pathway through which Internet use by older adults in China enhances their health status.

## 6 Conclusions and recommendations

Global efforts are underway to achieve healthy aging, with increasing attention focused on the health of older adults. In this study, a chain mediation model was constructed to empirically examine the health status of older adults, incorporating variables such as Internet use, social interaction, and physical exercise. The results showed that Internet use among older adults not only directly affects their health status but, more importantly, indirectly affects them through three critical paths: (1) indirectly improves health status by facilitating social interaction; (2) indirectly improves health status by facilitating physical exercise; and (3) ultimately improves health status by facilitating social interaction and then increasing physical exercise. This chain-mediated path of ‘Internet use → social interaction → physical exercise → health status’ has a significant effect.”

Based on these findings, the following recommendations are proposed:

First, internet use is a significant predictor of positive health status for the older adults. Therefore, it is imperative to increase internet usage among this population. However, a significant “digital divide” persists in China, where 53.4% of surveyed older adults individuals reported no prior internet usage. Previous research has shown that age, region, and educational level significantly affect internet use among the older adults (65). Specifically, internet usage decreases with age, is lower among those with less education, and is less common in China’s central, western, and rural regions. To address this, the government should promote internet adoption among the older adults and enhance internet public service infrastructure in central, western, and rural areas, narrowing the urban–rural digital divide. Additionally, the government should collaborate with community centers, older adults universities, and public libraries to offer large-scale, inclusive, and ongoing digital skills training, focusing on rural and low-income older adults.
Second, social interaction and physical exercise play a partially mediating role as well as a sequential chain mediating role between Internet use and the health status of Chinese older adults, and we propose the following policy recommendations. Develop age-friendly apps that focus on social interaction and sports, including online exercise courses and online communities for senior sports, to help older adults make connections, share exercise experiences, organize companion exercise, and enhance motivation to exercise. These apps can motivate older persons to participate in physical activity by launching online fitness challenges, signing up for rewards, and organizing offline sports events. This initiative strengthens social connections among older persons and promotes regular exercise, which is essential for overall health and well-being. The government should encourage relevant industries to develop such apps and provide financial support and policy incentives. At the same time, communities should be actively involved in promoting and organizing training activities to help older persons become familiar with and use these apps. In addition, policymakers should emphasize the potential of the Internet in health promotion for older persons and promote the integration of Internet services with geriatric health policies so as to form a model of health promotion that combines online and offline and create a healthier and more active living environment for older persons.

## 7 Limitation

This study is not without limitations. First, the data used for analysis are cross-sectional and thus cannot strictly establish causal relationships. Second, Internet use among older adults was measured only by its frequency, which does not adequately capture the diversity of Internet use, such as usage time and types of content accessed. At the same time, with regard to the measurement of health status variables, only the subjective assessment of health status by older persons was used, and in the future further combinations of BMI, ADL index or other objective health status variables with subjective variables will be carried out. Furthermore, although the study included several variables affecting the health status of older adults, it did not comprehensively analyze additional micro-level variables that may influence Internet use and health status. Consequently, future studies should employ longitudinal analyses and integrate qualitative methodologies to comprehensively explore the impact of Internet use on the health status of older adults and its underlying mechanisms. This will contribute to the current body of knowledge, offer a deeper and more comprehensive insight into the subject, and establish a foundation for subsequent research in related fields.

## Data Availability

Publicly available datasets were analyzed in this study. This data can be found here: http://cgss.ruc.edu.cn/.

## References

[1] Chen K Chan AHS. Predictors of gerontechnology acceptance by older Hong Kong Chinese. Technovation. (2014) 34:126–35. doi: 10.1016/j.technovation.2013.09.010

[2] Bao J Zhou L Liu G Tang J Lu X Cheng C. Current state of care for the elderly in China in the context of an aging population. Biosci Trends. (2022) 16:107–18. doi: 10.5582/bst.2022.01068, PMID: 35431289

[3] Zhu L Zhang Q Jing L Zhang X Wang F Zhang P. The relationship between loneliness and social care and health self-assessment among older adults in the community. Chin J Gerontol. (2018) 38:3238–40. doi: 10.3969/j.issn.1005-9202.2018.13.066

[4] Hu FF Zhang J Gao ZR Hong Z Xu LZ. Relationship between physical exercise and mental health among the urban and rural community's younger elderly. Chin Ment Health J. (2021) 35:739–44. doi: 10.3969/j.issn.1000-6729.2021.09.007

[5] China Internet Network Information Center. The 54th China internet development status report [Internet]. Beijing: CNNIC; (2024). Available online at:http://www.doc88.com/p-03473982557767.html

[6] Aggarwal B Xiong Q Schroeder-Butterfill E. Impact of the use of the internet on quality of life in older adults: review of literature. Prim Health Care Res Dev. (2020) 21:e55. doi: 10.1017/S1463423620000584, PMID: 33263273 PMC7737194

[7] Porter CE Donthu N. Using the technology acceptance model to explain how attitudes determine internet usage: the role of perceived access barriers and demographics. J Bus Res. (2006) 59:999–1007. doi: 10.1016/j.jbusres.2006.06.003

[8] Cobb S. Social support as a moderator of life stress. Psychosom Med. (1976) 38:300–14. doi: 10.1097/00006842-197609000-00003, PMID: 981490

[9] Kearns A Whitley E. Associations of internet access with social integration, well-being, and physical activity among adults in deprived communities: evidence from a household survey. BMC Public Health. (2019) 19:860. doi: 10.1186/s12889-019-7199-x, PMID: 31266470 PMC6604194

[10] Chen H Zhang T Li Y Zhao W Xu W. Relationship and mechanisms between internet use and physical exercise among middle-and younger-aged groups. PLoS One. (2024) 19:e0305131. doi: 10.1371/journal.pone.0305131, PMID: 38959189 PMC11221648

[11] Bangsbo J Blackwell J Boraxbekk CJ Caserotti P dela F Evans AB. Copenhagen consensus statement 2019: physical activity and ageing. Br J Sports Med. (2019) 53:856–8. doi: 10.1136/bjsports-2018-100451, PMID: 30792257 PMC6613739

[12] Nie P Nimrod G Sousa-Poza A. Internet use and subjective well-being in China. Soc Indic Res. (2017) 132:489–516. doi: 10.1007/s11205-016-1305-6

[13] McMellon CA Schiffman LG. Cybersenior mobility: why some older consumers may be adopting the internet. Adv Consum Res. (2000) 27:139.

[14] Yuan H. Internet use and mental health problems among older people in Shanghai, China: the moderating roles of chronic diseases and household income. Aging Ment Health. (2020) 25:657–63. doi: 10.1080/13607863.2020.1711858, PMID: 31928208

[15] Cotten SR Ford G Ford S Hale TM. Internet use and depression among retired older adults in the United States: a longitudinal analysis. J Gerontol B Psychol Sci Soc Sci. (2014) 69:763–71. doi: 10.1093/geronb/gbt051 24671896

[16] Erickson J Johnson GM. Internet Use and Psychological Wellness during Late Adulthood. Canadian Journal on Aging / La Revue canadienne du vieillissement (2011) 30:197–209. doi: 10.1017/S0714980811000109 24650669

[17] Meischke H Eisenberg M Rowe S Cagle A. Do older adults use the internet for information on heart attacks? Results from a survey of seniors in King County, Washington. Heart Lung. (2005) 34:3–12. doi: 10.1016/j.hrtlng.2004.06.006 15647729

[18] Xavier AJ d’Orsi E Wardle J Demakakos P Smith SG von Wagner C. Internet use and cancer-preventive behaviors in older adults: findings from a longitudinal cohort study. Cancer Epidemiol Biomarkers Prev. (2013) 22:2066–74. doi: 10.1158/1055-9965.EPI-13-0542 24148971

[19] Hamer M Stamatakis E. Prospective study of sedentary behavior, risk of depression, and cognitive impairment. Med Sci Sports Exerc. (2014) 46:718–23. doi: 10.1249/MSS.0000000000000156, PMID: 24121248 PMC3960356

[20] Peng YI Chan YS. Do internet users lead a healthier lifestyle? J Appl Gerontol. (2020) 39:277–83. doi: 10.1177/0733464819866849 29957087

[21] Liang L. Social media use, social interaction in reality and youth health——empirical analysis based on CGSS data. Int J Front Sociol. (2022) 4:115–21. doi: 10.25236/IJFS.2022.041020

[22] Glei DA Landau DA Goldman N Chuang YL Rodríguez G Weinstein M. Participating in social activities helps preserve cognitive function: an analysis of a longitudinal, population-based study of the elderly. Int J Epidemiol. (2005) 34:864–71. doi: 10.1093/ije/dyi049 15764689

[23] Chiao C Weng LJ Botticello AL. Social participation reduces depressive symptoms among older adults: an 18-year longitudinal analysis in Taiwan. BMC Public Health. (2011) 11:292. doi: 10.1186/1471-2458-11-292 21569285 PMC3103460

[24] Yuasa M Ukawa S Ikeno T Kawabata T. Multilevel, cross-sectional study on social capital with psychogeriatric health among older Japanese people dwelling in rural areas. Australas J Ageing. (2014) 33:E13–9. doi: 10.1111/ajag.12024 24520916

[25] Humphreys BR McLeod L Ruseski JE. Physical activity and health outcomes: evidence from Canada. Health Econ. (2014) 23:33–54. doi: 10.1002/hec.2900 23364850

[26] Wang H Shen B Bo J. Profiles of health-related quality of life and their relationships with happiness, physical activity, and fitness. Res Q Exerc Sport. (2022) 93:260–9. doi: 10.1080/02701367.2020.1822985 33030420

[27] Leith LM Taylor AH. Psychological aspects of exercise: a decade literature review. J Sport Behav. (1990) 13:219.

[28] Li N. Effect of physical exercise on self-rated health and physical fitness status in the elderly. Journal of Sports Science (2010) 31:84–87. doi: 10.3969/j.issn.1004-4590.2010.01.021

[29] Nakagomi A Shiba K Kawachi I Kondo K. Internet use and subsequent health and wellbeing in older adults: an outcome-wide analysis. Comput Human Behav. (2022) 130:107156. doi: 10.1016/j.chb.2021.107156, PMID: 40690924

[30] Kraut R Patterson M Lundmark V Kiesler S Mukophadhyay T Scherlis W. Internet paradox: a social technology that reduces social involvement and psychological well-being? Am Psychol. (1998) 53:1017–31. doi: 10.1037/0003-066X.53.9.1017, PMID: 9841579

[31] Fenelon C Murphy EP Kearns SR Curtin W Murphy CG. A growing challenge: the rise of femoral periprosthetic fractures–an 11-year observational study. Surgeon. (2020) 18:19–23. doi: 10.1016/j.surge.2019.05.001 31196725

[32] Agbangla NF Séba MP Bunlon F Toulotte C Fraser SA. Effects of physical activity on physical and mental health of older adults living in care settings: a systematic review of meta-analyses. Int J Environ Res Public Health. (2023) 20:6226. doi: 10.3390/ijerph20136226, PMID: 37444074 PMC10341127

[33] Duplaga M. The association between internet use and health-related outcomes in older adults and the elderly: a cross-sectional study. BMC Med Inform Decis Mak. (2021) 21:150. doi: 10.1186/s12911-021-01500-2, PMID: 33957909 PMC8100743

[34] Durlauf SN Ioannides YM. Social interactions. Annu Rev Econ. (2010) 2:451–78. doi: 10.1146/annurev.economics.050708.143312

[35] Loprinzi PD Joyner C. Source and size of emotional and financial-related social support network on physical activity behavior among older adults. J Phys Act Health. (2016) 13:776–9. doi: 10.1123/jpah.2015-0629 26900842

[36] Narkauskaitė L Samsonienė L Karanauskienė D Stankutė V. Psychomotor abilities of elderly people and their motivation to participate in organized physical activity. Exp Aging Res. (2020) 46:257–71. doi: 10.1080/0361073X.2020.1743614 32194001

[37] Shephard RJ. Exercise for the frail elderly. Res Sports Med. (1990) 1:263–77. doi: 10.1080/15438629009511884, PMID: 40640861

[38] Tiebout CM. A pure theory of local expenditures. J Polit Econ. (1956) 64:416–24. doi: 10.1086/257839

[39] Liu XH Yang F Wang XD. Related factors and equity of health status among floating population in China based on geographic information system analysis. J Peking University (Med). (2024) 56:223–9. doi: 10.19723/j.issn.1671-167X.2024.02.004, PMID: 38595237 PMC11004974

[40] Yang J Niu L Lu C. The impact of internet use on health status among older adults in China: the mediating role of social support. Front Public Health. (2023) 11:1108096. doi: 10.3389/fpubh.2023.1108096, PMID: 36908418 PMC9992883

[41] Jiang Q Chen Z. Active aging of silver-haired surfers: internet use enhances a study on the mechanism of the role of subjective well-being of the elderly. Mod Commun. (2021) 43:41–8. doi: 10.19997/j.cnki.xdcb.2021.12.007

[42] Dong X Meng S Chen D. How does the internet enhance the subjective well-being of elderly individuals in China? Front Psychol. (2022) 13:1036169. doi: 10.3389/fpsyg.2022.1036169, PMID: 36329732 PMC9622754

[43] Zhang M Yuan L Ke Z Jian J Tan H Lv G. How does social insurance affect the social interactions of rural residents in China: study on the impact of rural formal social security system on informal social security mechanism. Front Psychol. (2022) 13:751946. doi: 10.3389/fpsyg.2022.751946, PMID: 35356356 PMC8959861

[44] Liu N Zhong Q. The impact of sports participation on individuals’ subjective well-being: the mediating role of class identity and health. Humanit Soc Sci Commun. (2023) 10:1–9. doi: 10.1057/s41599-023-01533-0

[45] Fang J Zhang MQ Qiu HZ. Mediation analysis and effect size measurement: retrospect and prospect. Psychol Dev Educ. (2012) 28:105–111.

[46] Hayes AF Process: a versatile computational tool for observed variable mediation, moderation, and conditional process modeling (2012) Available online at: http://www.afhayes.com/public/process2012.pdf

[47] Amini R Chee KH Mendieta M Parker S. Online engagement and cognitive function among older adults. Geriatr Gerontol Int. (2019) 19:918–23. doi: 10.1111/ggi.13749, PMID: 31368165

[48] Szulc K Duplaga M. The association between internet use and health outcomes in the population of older adults. Eur J Pub Health. (2020) 30:cka165.443. doi: 10.1093/eurpub/ckaa165.443

[49] Chang X Ni X Chun Z Meng Z. Influence of internet use on commercial health insurance of Chinese residents. Front Public Health. (2022) 10:907124. doi: 10.3389/fpubh.2022.907124 35899171 PMC9311374

[50] Zhang J Cheng M Mei R Wang F. Internet use and individuals’ environmental quality evaluation: evidence from China. Sci Total Environ. (2020) 710:136290. doi: 10.1016/j.scitotenv.2019.136290, PMID: 31923668

[51] Wong CK Yeung DY Ho HC Wong CKM Ho HCY Tse K-P. Chinese older adults' internet use for health information. J Appl Gerontol. (2014) 33:316–35. doi: 10.1177/0733464812463430, PMID: 24717738

[52] Zhao Y Zhao M Song S. Online health information seeking behaviors among older adults: systematic scoping review. J Med Internet Res. (2022) 24:e34790. doi: 10.2196/34790, PMID: 35171099 PMC8892316

[53] Cohall AT Nye A Moon-Howard J Kukafka R Dye B Vaughan RD. Computer use, internet access, and online health searching among Harlem adults. Am J Health Promot. (2011) 25:325–33. doi: 10.4278/ajhp.090325-QUAN-121, PMID: 21534835

[54] Hou P. Influence mechanism of internet use on the physical and mental health of the Chinese elderly—based on Chinese general social survey. PLoS One. (2025) 20:e0312664. doi: 10.1371/journal.pone.0312664, PMID: 39752532 PMC11698357

[55] Cotten SR Ford G Ford S Hale TM. Internet use and depression among older adults. Comput Human Behav. (2012) 28:496–9. doi: 10.1016/j.chb.2011.10.023

[56] Lelkes O. Happier and less isolated: internet use in old age. J Poverty Soc Justice. (2013) 21:33–46. doi: 10.1332/175982713X664047

[57] Christine D Eva H Andreas M. Efficacy of internet-based interventions for common mental disorder symptoms and psychosocial problems in older adults: a systematic review and meta-analysis. Internet Interv. (2022) 27:100498. doi: 10.1016/j.invent.2022.100498 35141136 PMC8810404

[58] Mouton A Cloes M. Older adults, physical activity and the internet: exploring their behaviors, beliefs and opinions. Int J Phys Educ. (2014) 51:18–29. doi: 10.5771/2747-6073-2014-1-18

[59] Parker K Brown HL Salmon J. Are there common correlates of adolescents' sport participation and screen time? Res Q Exerc Sport. (2023) 94:374–82. doi: 10.1080/02701367.2021.1998305, PMID: 35352992

[60] Sasaki S Sato A Tanabe Y Matsuoka S Adachi A Kayano T. Internet use and physical activity of older adults during the COVID-19 pandemic: a cross-sectional study in a northern Japanese City. BMC Geriatr. (2022) 22:688. doi: 10.1186/s12877-022-03360-5, PMID: 35986245 PMC9390958

[61] Fan Y Hu Y Wang F. Physical exercises of Chinese older adults and social participation: health promotion and network expansion. Popul Res. (2021) 45:97–113.

[62] Wellman B Quan-Haase A Boase J Chen W Hampton K Díaz I. The social affordances of the internet for networked individualism. J Comput-Mediat Commun. (2003) 8:JCMC834. doi: 10.1111/j.1083-6101.2003.tb00216.x, PMID: 40693313

[63] Dalen HB Seippel O. Friends in sports: social networks in leisure, school and social media. Int J Environ Res Public Health. (2021) 18:6197. doi: 10.3390/ijerph18126197, PMID: 34201132 PMC8229858

[64] Nascimento H Martinez-Perez C Alvarez-Peregrina C Sanchez-Tena MA. The role of social Media in Sports Vision. Int J Environ Res Public Health. (2021) 18:5354. doi: 10.3390/ijerph18105354, PMID: 34069821 PMC8157247

[65] Wu J Wang YM. Research on the current status and influencing factors of internet use among the elderly in China—an analysis based on the 2017 CGSS data. J Aging Sci Res. (2021) 9:43–5. doi: 10.3969/j.issn.2095-5898.2021.09.005

